# Patient reported clinical outcomes: the challenges and implications for randomised controlled trials

**DOI:** 10.1186/1745-6215-12-S1-A72

**Published:** 2011-12-13

**Authors:** Suzanne Breeman, Alison McDonald, Gladys McPherson, Graeme MacLennan, Marion Campbell, Kath Starr, Seonaidh Cotton

**Affiliations:** 1 Health Services Research Unit, University of Aberdeen, Aberdeen, AB25 2ZD, UK

## Objectives

Clinical outcomes are an important component of randomised controlled trials (RCTs) and are often used to complement patient reported outcomes measures such as health-related quality of life. Although clinical outcomes were traditionally collected through clinical examination or laboratory results, routine data sources and self-reporting by patients are now being increasingly used. We describe patient reported clinical outcomes and the challenges associated with collection of data in this way.

## Methods

Four RCTs that collected patient reported clinical outcomes through postal questionnaires were examined. In each RCT the patient reported clinical outcome was verified using either medical records, routine data sources or by contacting the patient’s general practitioner or Consultant to ascertain the accuracy of reporting by the patient.

## Results

The accuracy of patient reporting of clinical outcomes is dependent on a number of factors, including the nature and timing of the clinical outcome and the phrasing of the clinical questions. For example, it may be easier for a patient to report a knee-related hospital re-admissions than self-report a urinary tract infection. Nevertheless, approximately 15% of patient reported knee-related hospital re-admission (collected through annual postal questionnaires) could not be verified through routine data sources and/or medical records. Such inconsistencies were shown to be a combination of misunderstanding by the patient and inaccuracies of the routine data sets.

## Conclusions

Obtaining clinical information from the perspective of the patient remains important, especially if the outcome of interest is a symptomatic one. However with the potential inaccuracies associated with patient reporting of clinical outcomes, it may be necessary to consider verifying such outcomes with medical professionals and/or routine data sources. Such a strategy has implications in terms of staff time and cost and therefore has to be considered during the design stage of the RCT.

We will discuss some inconsistencies between self-reporting and medically confirmed clinical outcomes. We will highlight processes involved in verifying patient reported clinical outcomes and how adopting such a verification strategy may impact on the overall trial results.

